# Supplementary material for: A Critical View of the Application of the APEX Software (Aqueous Photochemistry of Environmentally-Occurring Xenobiotics) to Predict Photoreaction Kinetics in Surface Freshwaters
Source: Molecules. 2019 Dec 18;25(1):9. doi: 10.3390/molecules25010009 (PMC7017383; doi:10.3390/molecules25010009)
Supplement: Supplementary file 1 [file molecules-25-00009-s001.zip › 3.2.4_gcc-4.4.0.zip!/3.2.4_gcc-4.4.0/3.2.4_gcc-4.4.0/doc/octave/html/interpreter/Real-Life-Example.html]

Real Life Example - Untitled


Previous: Iterative Techniques,
Up: Sparse Matrices


---

### 21.4 Real Life Example of the use of Sparse Matrices

A common application for sparse matrices is in the solution of Finite
Element Models. Finite element models allow numerical solution of
partial differential equations that do not have closed form solutions,
typically because of the complex shape of the domain.

In order to motivate this application, we consider the boundary value
Laplace equation. This system can model scalar potential fields, such
as heat or electrical potential. Given a medium
Omega
with boundary
dOmega
. At all points on the
dOmega
the boundary conditions are known, and we wish to calculate the potential in
Omega
. Boundary conditions may specify the potential (Dirichlet
boundary condition), its normal derivative across the boundary
(Neumann boundary condition), or a weighted sum of the potential and
its derivative (Cauchy boundary condition).

In a thermal model, we want to calculate the temperature in
Omega
and know the boundary temperature (Dirichlet condition)
or heat flux (from which we can calculate the Neumann condition
by dividing by the thermal conductivity at the boundary). Similarly,
in an electrical model, we want to calculate the voltage in
Omega
and know the boundary voltage (Dirichlet) or current
(Neumann condition after diving by the electrical conductivity).
In an electrical model, it is common for much of the boundary
to be electrically isolated; this is a Neumann boundary condition
with the current equal to zero.

The simplest finite element models will divide
Omega
into simplexes (triangles in 2D, pyramids in 3D).
We take as an 3D example a cylindrical liquid filled tank with a small
non-conductive ball from the EIDORS project^1^. This is model is designed to reflect
an application of electrical impedance tomography, where current patterns
are applied to such a tank in order to image the internal conductivity
distribution. In order to describe the FEM geometry, we have a matrix of
vertices `nodes` and simplices `elems`.

The following example creates a simple rectangular 2D electrically
conductive medium with 10 V and 20 V imposed on opposite sides
(Dirichlet boundary conditions). All other edges are electrically
isolated.

```
        node_y= [1;1.2;1.5;1.8;2]*ones(1,11);
        node_x= ones(5,1)*[1,1.05,1.1,1.2, ...
                  1.3,1.5,1.7,1.8,1.9,1.95,2];
        nodes= [node_x(:), node_y(:)];
     
        [h,w]= size(node_x);
        elems= [];
        for idx= 1:w-1
          widx= (idx-1)*h;
          elems= [elems; ...
            widx+[(1:h-1);(2:h);h+(1:h-1)]'; ...
            widx+[(2:h);h+(2:h);h+(1:h-1)]' ];
        endfor
     
        E= size(elems,1); # No. of simplices
        N= size(nodes,1); # No. of vertices
        D= size(elems,2); # dimensions+1
```

This creates a N-by-2 matrix `nodes` and a E-by-3 matrix
`elems` with values, which define finite element triangles:

```
       nodes(1:7,:)'
         1.00 1.00 1.00 1.00 1.00 1.05 1.05 ...
         1.00 1.20 1.50 1.80 2.00 1.00 1.20 ...
     
       elems(1:7,:)'
         1    2    3    4    2    3    4 ...
         2    3    4    5    7    8    9 ...
         6    7    8    9    6    7    8 ...
```

Using a first order FEM, we approximate the electrical conductivity
distribution in
Omega
as constant on each simplex (represented by the vector `conductivity`).
Based on the finite element geometry, we first calculate a system (or
stiffness) matrix for each simplex (represented as 3-by-3 elements on the
diagonal of the element-wise system matrix `SE`. Based on `SE`
and a N-by-DE connectivity matrix `C`, representing the connections
between simplices and vertices, the global connectivity matrix `S` is
calculated.

```
       # Element conductivity
       conductivity= [1*ones(1,16), ...
              2*ones(1,48), 1*ones(1,16)];
     
       # Connectivity matrix
       C = sparse ((1:D*E), reshape (elems', ...
              D*E, 1), 1, D*E, N);
     
       # Calculate system matrix
       Siidx = floor ([0:D*E-1]'/D) * D * ...
              ones(1,D) + ones(D*E,1)*(1:D) ;
       Sjidx = [1:D*E]'*ones(1,D);
       Sdata = zeros(D*E,D);
       dfact = factorial(D-1);
       for j=1:E
          a = inv([ones(D,1), ...
              nodes(elems(j,:), :)]);
          const = conductivity(j) * 2 / ...
              dfact / abs(det(a));
          Sdata(D*(j-1)+(1:D),:) = const * ...
              a(2:D,:)' * a(2:D,:);
       endfor
       # Element-wise system matrix
       SE= sparse(Siidx,Sjidx,Sdata);
       # Global system matrix
       S= C'* SE *C;
```

The system matrix acts like the conductivity
`S`
in Ohm's law
`S * V = I`.
Based on the Dirichlet and Neumann boundary conditions, we are able to
solve for the voltages at each vertex `V`.

```
       # Dirichlet boundary conditions
       D_nodes=[1:5, 51:55];
       D_value=[10*ones(1,5), 20*ones(1,5)];
     
       V= zeros(N,1);
       V(D_nodes) = D_value;
       idx = 1:N; # vertices without Dirichlet
                  # boundary condns
       idx(D_nodes) = [];
     
       # Neumann boundary conditions.  Note that
       # N_value must be normalized by the
       # boundary length and element conductivity
       N_nodes=[];
       N_value=[];
     
       Q = zeros(N,1);
       Q(N_nodes) = N_value;
     
       V(idx) = S(idx,idx) \ ( Q(idx) - ...
                 S(idx,D_nodes) * V(D_nodes));
```

Finally, in order to display the solution, we show each solved voltage
value in the z-axis for each simplex vertex.
See fig:femmodel.

```
       elemx = elems(:,[1,2,3,1])';
       xelems = reshape (nodes(elemx, 1), 4, E);
       yelems = reshape (nodes(elemx, 2), 4, E);
       velems = reshape (V(elemx), 4, E);
       plot3 (xelems,yelems,velems,'k');
       print ('grid.eps');
```

**Figure 21.6: Example finite element model the showing triangular elements.
The height of each vertex corresponds to the solution value.**


---

#### Footnotes

[1] EIDORS - Electrical
Impedance Tomography and Diffuse optical Tomography Reconstruction Software
http://eidors3d.sourceforge.net

---
